# Intranasal inoculation with *Bordetella pertussis* confers protection without inducing classical whooping cough in baboons

**DOI:** 10.1016/j.crmicr.2021.100072

**Published:** 2021-10-09

**Authors:** Thibaut Naninck, Vanessa Contreras, Loïc Coutte, Sébastien Langlois, Aurélie Hébert-Ribon, Magali Pelletier, Nathalie Reveneau, Camille Locht, Catherine Chapon, Roger Le Grand

**Affiliations:** a Université Paris-Saclay, Inserm, CEA, Center for Immunology of Viral, Auto-immune, Hematological and Bacterial Diseases (IMVA-HB/IDMIT), Fontenay-aux-Roses & Le Kremlin-Bicêtre, France; b Univ. Lille, CNRS, Inserm, CHU Lille, Institut Pasteur de Lille, U1019 – UMR8204 – CIIL - Center for Infection and Immunity of Lille, F-59000 Lille, France; c Sanofi-Pasteur MSD, Marcy l'Etoile, France

**Keywords:** Whooping cough, Pertussis, Non-human primates, Baboons

## Abstract

**Background:**

The resurgence of whooping cough in many countries highlights the crucial need for a better understanding of the pathogenesis of respiratory infection by *Bordetella pertussis*. Exposure of baboons to *B. pertussis* by the intranasal and intra-tracheal routes is a recently described preclinical model that reproduces both *B. pertussis* infection of humans and whooping cough disease. Here, we tested both intranasal and intranasal+intra-tracheal exposure routes and assessed their impact on disease development and immunity.

**Methods:**

Young baboons were intranasally exposed to the B1917 clinical isolate, representative of circulating strains in Europe, or its green-fluorescent protein expressing derivative. Animals were followed for pertussis symptoms and bacterial colonization and by *in vivo* probe-based confocal laser endomicroscopy (pCLE) imaging. Sero-conversion and protection against subsequent infection were then evaluated.

**Results:**

Seroconversion and bacterial colonization of both the nasopharynx and trachea was observed in baboons exposed to B. pertussis by the intranasal route only, and also in those animals challenged by both the intranasal and intra-tracheal routes together. However, baboons exposed solely by the intranasal route developed only mild clinical symptoms, with no paroxysmal cough. These animals were protected against re-infection by *B. pertussis*.

**Conclusions:**

Intranasal exposure of baboons to *B. pertussis* does not induce disease but elicits immune mechanisms that protect them from subsequent exposure to the bacteria. These findings suggest that the intranasal route of inoculation in this non-human primate model could be used in the pre-clinical evaluation of nasal candidate vaccines against pertussis.

## Highlights

• In this manuscript, we describe the impact of *Bordetella pertussis* exposure route on whooping cough pathogenesis in baboons. We demonstrate in this paper that intranasal exposure of animals with a clinical isolate (or its fluorescent derivative) of *B. pertussis* induced classical nasopharyngeal and tracheal colonization but without inducing pertussis symptoms (cough and leukocytosis) compared to animals exposed to the classical combined intranasal and intra-tracheal routes with the same bacterial strains. Moreover, this intranasal exposure induces good *B. pertussis* specific seroconversion and provides protection from further infection.

## Introduction

Pertussis, or whooping cough, is mainly caused by *Bordetella pertussis* infection of human airways. Despite high global vaccination coverage with diphtheria-tetanus-pertussis vaccines (85% in 2019 (“WHO | World Health Organization,” n.d.)), this disease still affects millions of young people (24.1 million cases in 2014) annually, leading to 160,700 deaths of children under-five in 2014 (Yeung et al., 2017). The reported frequency of the disease has significantly increased over the last 10 years, even in several high-income countries with excellent vaccine coverage (Locht, 2016). The reason for such a resurgence is still a matter of debate and may include better diagnosis of *B. pertussis.* infections due to enhanced surveillance and improved diagnostic tools, strain adaptations, waning of immunity after the introduction of acellular vaccines (aP), and asymptomatic carriage, which favors *B. pertussis* circulation (Locht, 2016).

Recently, the development of a non-human primate (NHP) model of pertussis has improved our understanding of the pathogenesis of whooping cough (Warfel et al., 2012). In contrast to other animal models, young baboons display all clinical symptoms of pertussis disease following *B. pertussis* exposure by combined intranasal and intra-tracheal (IN+IT) routes, including paroxysmal cough and leukocytosis (Warfel et al., 2012). However, the combined administration route may not fully reflect natural transmission, as direct exposure of the trachea may bypass critical innate and adaptive immune effectors present in the nasal cavity. This may also be relevant for future evaluation of the efficacy of new pertussis vaccines (Jahnmatz et al., 2020; Locht, 2017, 2016). In addition, a controlled infection by experimental exposure of human volunteers to the same B1917 *B. pertussis.* strain has been recently reported (de Graaf et al., 2017). We consider the predictive value of the Baboon model to be highly relevant for the comparison of infection in the two species using a similar approach.

We aimed to compare the effect of *B. pertussis* exposure by the IN route only *versus* the combined IN and IT routes on the clinical symptoms of whooping cough, bacterial colonization, and immune responses in young baboons. We also aimed to evaluate protection against subsequent *B. pertussis* re-infection of convalescent animals previously exposed by either of the two approaches.

## Materials and methods

### Ethics statement

All studies were performed on juvenile *Papio Anubis* (10–16 months) obtained from the *Station de Primatologie* (CNRS, Rousset-sur-Arc, France) and then housed in the facilities of the Infectious Disease Models and Innovative Therapies (IDMIT) center at the “Commissariat à l'Energie Atomique et aux Energies Alternatives” (CEA, Fontenay-aux-Roses, France). The animals were used in accordance with French national regulations (facilities authorization Number D92-032-02). The CEA complies with the Standards for Humane Care and Use of Laboratory Animals of the Office for Laboratory Animal Welfare (OLAW, USA) under OLAW Assurance number #A5826–01. The use of NHPs at the CEA complies with the recommendations of European Directive 2010/63 (recommendation N°9). These experiments were approved by the local ethics committee number 44 (statement number A16-041) and approved by the French Ministry of Research and Education (APAFIS#6290–2016071816091539 v3). Experimental procedures (animal handling, bacterial inoculation, and blood sampling) were conducted after sedating the animals with Domitor (Vetoquinol, France, 0.05 mL/kg) and ketamine hydrochloride (Rhône-Mérieux, France, 10 mg/kg). After the experimental procedure, baboons were injected with Antisedan (Vetoquinol, France, 0.05 mL/kg) to facilitate recovery from sedation. For the collection of tissues at necropsy, animals were anesthetized with ketamine and then euthanized by intravenous injection of 180 mg/kg sodium pentobarbital.

### Bacterial strains and growth media

The *B. pertussis* clinical isolate B1917 was provided by Frits Mooi and came from the RIVM collection (Bilthoven, The Netherlands). This strain is considered representative of a predominant global lineage of *B. pertussis* with the genotype pertussis toxin promoter allele 3, pertussis toxin subunit A allele 1 (ptxP3-ptxA1) (Bart et al., 2014).

B1917 iGFP carries an insertion of a 934-bp DNA fragment containing Ppor (promoter of *bp0840*), which drives the expression of the green fluorescent protein (GFP) gene inserted within the chromosomal *ureB* locus. It was obtained as follows. Two DNA fragments flanking the *ureB* locus were obtained by PCR using genomic DNA from *B. pertussis* BPSM (Menozzi et al., 1994) as template and the oligonucleotide pairs 5′-TATACTCGAGTGCGAGCTCGAACTG-3′ and 5′-TATAGGTACCGTCGAGAAGGACCACAC-3′, and 5′- TATAGAATTCGACACCTCCCTGGTCAGACC-3′ and 5′-TATAGGATCCCCGCCCTGATGCTGTTCTCG-3′ as primers. The resulting *Xho*I*-Kpn*I and *Eco*RI*-Bam*HI fragments were then introduced in two steps into pSS4940 (a pSS4245-based suicide plasmid (Inatsuka et al., 2010)), yielding pSS4940 UreB. The DNA fragment carrying Ppor-*gfp* was obtained by PCR using pBBPG (Hibrand-Saint Oyant et al., 2005) as template and the oligonucleotide pair 5′-TATAGGTACCTCGAGCCCGCGCGCGATTCCGGAT-3′ and 5′-TATAGAATTCCTATTTGTATAGTTCATCCATGCC-3′ as primers. The resulting *Kpn*I*-Eco*RI fragment was then introduced into pSS4940 UreB, yielding pSS4940 UreB::Ppor-GFP. This construct was electroporated (200 ng of DNA at 2.5 kV, 600 Ω, 25µF) into B1917 and used for allelic exchange, yielding B1917 iGFP.

*B. pertussis* strains were grown on Bordet-Gengou agar plates containing 10% (v/v) sheep blood (BG plates, BD) for 72 h at 37 °C.

For growth studies, bacteria were cultivated at 37 °C, as described in Locht et al., in modified Stainer-Scholte medium (Locht et al., 1992) containing cyclodextrin (1 g/L) and casamino acids (5 g/L). The optical density OD_600nm_ was measured every minute to evaluate bacterial growth using an EloCheck Quatro device (biotronix GmbH). Cultures were performed in duplicate.

### Preparation of inoculum and infection of baboons

*B. pertussis* colonies (B1917 or B1917-iGFP) grown on BG plates for 72 h at 37 °C were resuspended in sterile phosphate-buffered saline (PBS) at an OD_600nm_ of 0.9, which corresponds to a density of 10^8^ to 10^9^ CFU/mL. Baboons were infected either by the IN+IT routes (control, *n* = 2), as previously described (Merkel and Halperin, 2014; Naninck et al., 2018; Warfel et al., 2012), or by the IN route only (*n* = 6). Intranasal + intra-tracheal exposure was performed introducing first 0.5 mL of the bacterial inoculum in each nostril via a catheter tubing connected to a 1 mL syringe and then 1 mL of the bacterial inoculum via a nasogastric tube inserted into the trachea and connected to a 2 mL syringe. Intranasal only exposure was performed exactly like the first step of the IN+IT route but using a twice concentrated inoculum. Overall, each animal was inoculated with the same total number of bacteria.

Four months after the first inoculation, a number of the animals were infected a second time by the IN+IT routes with the same inoculum dose and the same bacterial strain (B1917 or B1917-iGFP) to evaluate the protection conferred by the first infection. The inoculum concentration was validated by serial dilutions and plating onto BG blood agar plates and colony counting after seven days of incubation at 37 °C. Automatic colony counting was performed using a Scan®300 colony counter (Interscience, France).

### Bacterial colonization and monitoring of infection

Bacterial load in the nasopharynx was measured twice weekly using flocked swabs in each nostril and then pooled to have and overall estimates of the nasal bacterial content. Bacterial load in the upper section of the trachea was measured weekly using flocked swabs in the trachea. Samples were then collected and resuspended in liquid Amies medium (COPAN, USA). This suspension (50 μL) was plated in duplicate in serial dilutions onto BG blood agar plates. The number of colony-forming units (CFU) per plate was estimated by automatic counting after seven days of incubation at 37 °C. Bacterial content in the lungs was estimated at baseline and before euthanasia of the animals by broncho-alveolar lavage (BAL) performed with 50 mL of PBS. Whole blood was evaluated for the number of circulating white blood cells and lymphocytes by a complete blood count.

### Serum IGG quantification by multiplex antibody assay

The quantification of total IgG anti-filamentous hemagglutinin (FHA) and anti-pertussis toxin (PT) was performed by a multiplex antibody assay as described previously (Cole et al., 2020). Briefly, each antigen was biotinylated and linked to unique spots on Meso Scale Discovery U-plex plates. Plates were coated with PT (5 µg/mL) and FHA (25 µg/mL). Serial diluted serum samples were added to plates on which the antigen-specific antibodies can bind to specific linkers. Detection was performed by the addition of antibodies conjugated to electrochemiluminescent labels (sulfo-tag-conjugated anti-HU/NHP IgG antibody from MSD). Plates were read by placing them into the MSD instrument, which measures the chemiluminescent signal. The MSD Discovery Workbench software was used to calculate the titer for each serum dilution (using a sigmoidal four-parameter curve) in reference to a titrated pool of Baboon sera, generated by immunization with aP vaccine. The final titer for a given serum sample was reported after calculating the geometric mean of titers obtained for each dilution of that serum sample.

### Assessment of the stability of *in vivo* fluorescence

The stability of *B. pertussis* B1917-iGFP fluorescence was assessed *in vivo* using bacteria recovered from infected baboon nasopharyngeal swabs. Briefly, bacteria from nasopharyngeal swab samples obtained from B1917-iGFP-infected baboons were cultivated for seven days on BG blood agar plates. Bacteria were stained with 4′6-diamidino-2-phenylindole (DAPI) for 20 min at room temperature and freshly observed by confocal microscopy. Images were acquired using a Nikon-A1R confocal laser-scanning microscope attached to an inverted ECLIPSE-Ti (Nikon, Japan). Ten representative fields were acquired at each time point and the percentage of GFP-positive *B. pertussis* assessed as the ratio between GFP and DAPI-stained bacteria.

### Quantification of coughing

Animal cages were monitored with a video-recording system. The occurrence of cough for each animal was assessed daily during two 3-h periods (6:00–9:00 a.m., 5:00–8:00 p.m.) using Windows Movie Maker software.

### *In vivo* pCLE imaging of the baboon respiratory tract

*In vivo* probe-based confocal laser endomicroscopy (pCLE) (Cellvizio®, Mauna Kea Technologies, France) with bronchoscopy (Karl Storz Special fibroscope) was performed as described (Naninck et al., 2018; Thiberville et al., 2007). Briefly, the optical miniZ probe was inserted into the working channel of the bronchoscope and imaging sequences captured from the top of the trachea down to the secondary bronchi. *B. pertussis* B1917-iGFP bacteria were detected at 488 nm. The background signal in this channel was assessed before baboon challenge, in the absence of fluorescent bacteria. *In vivo* imaging sequences were captured once a week for four weeks, starting seven days before infecting the animals with *B. pertussis*.

### Image analysis

Movies obtained using pCLE were first selected using IC-Viewer software and then exported to ImageJ software (National Institute of Mental Health, USA). Intensity thresholds were fixed to minimize the background to signal ratio. Bacterial colonization of the lower airways was assessed by GFP area quantification. Briefly, the total area of GFP-positive signal was calculated for each imaging time-point using ImageJ Software in 30 images randomly extracted from the pCLE acquisition. No signal size restriction was applied due to the small size of the bacteria.

### Statistical analysis

Data are reported as the means ± standard errors for replicate experiments (GraphPad Prism software).

### Data availability

All data generated or analyzed during these studies are available from the corresponding author.

## Results

### Generation and characterization of *B. pertussis* B1917-iGFP

The B1917 fluorescent derivative B1917-iGFP was analyzed by PCR and found to contain the *ptxA1, prn-2*, and *fim3–2* alleles, as described for the parental strain B1917 (Bart et al., 2014).

**Fig. 1:**
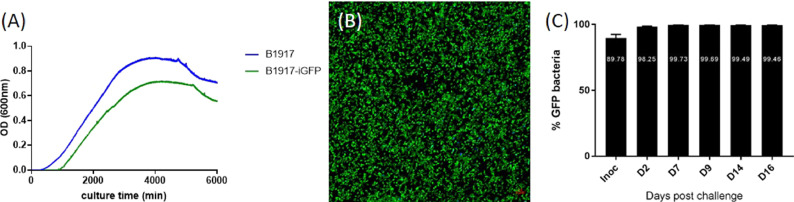
Characterization of B. pertussis B1917-iGFP. (A) Comparison of the growth rates between B1917 (blue curve) and B1917-iGFP (green curve) in liquid culture. (B) Fluorescence analysis by confocal microscopy (GFP in green and DAPI in blue). (C) Percentage of GFP-expressing bacteria over time after infection of the baboons.

We compared the growth of B1917 and B1917-iGFP in liquid culture before the use of B1917-iGFP for *in vivo* infection. The growth rate of the GFP-producing bacteria was slightly lower than that of the parental strain (Fig. 1A). Fluorescence of the bacteria freshly grown on BG blood agar plates was assessed by confocal microscopy. *B. pertussis* B1917-iGFP showed an intense bright signal after excitation with a 488-nm laser (Fig. 1B).

The stability of the *in vivo* fluorescence was assessed on bacteria recovered from nasopharyngeal swabs of juvenile baboons at various time points after IN exposure to B1917-iGFP. Between 98.2 and 99.8% of all *B. pertussis* recovered from baboon nasal swab samples was found to be GFP-positive by confocal microscopy at every time point up to 16 days post-infection (Fig. 1C).

### Intranasal inoculation of *B. pertussis* induces infection with mild clinical symptoms in baboons

**Fig. 2:**
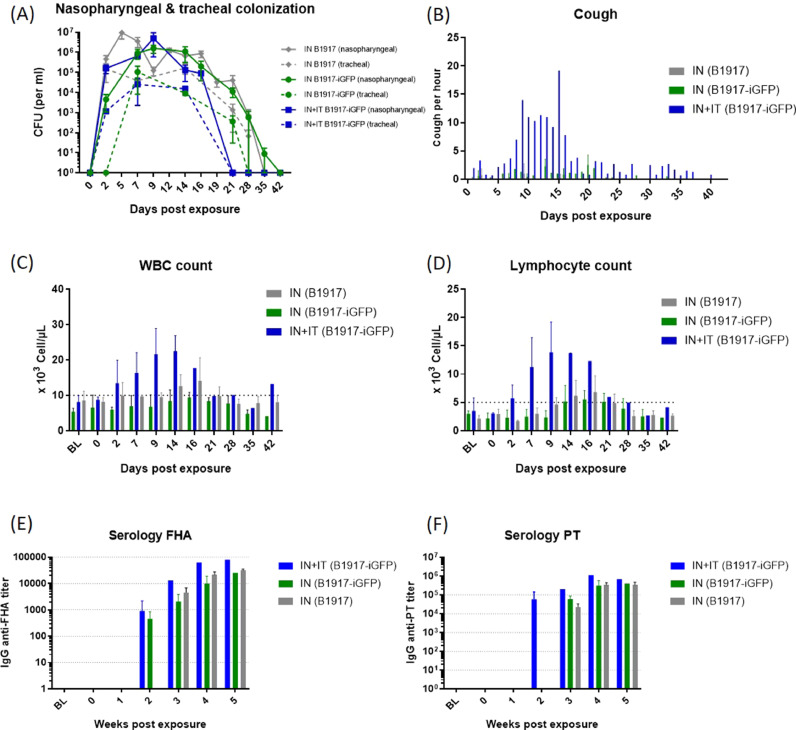
Clinical symptoms of pertussis in baboons after B1917 or B1917-iGFP challenge by the intranasal (IN) only (gray & green curves) or intranasal or intratracheal (IN+IT) routes (blue curves). (A) Nasopharyngeal and tracheal B. pertussis colonization was estimated by plating swabs on Bordet Gengou blood agar plates. (B) Episodes of paroxysmal cough recorded per hour. (C) Circulating white blood cell numbers, (D) lymphocyte numbers (D), and anti-FHA IgG (E) and anti-PT IgG (F) titers were determined over time.

We assessed the impact of the inoculation route on infection by *B. pertussis* by exposing juvenile baboons, aged of 10 to 16 months, to 10^7^–10^8^ CFU of B1917-iGFP or B1917, either using the combined IN+IT route (*n* = 2) or the IN route only (*n* = 6) (Groups described in Table 1). All animals showed nasopharyngeal bacterial colonization starting from day 2 post-challenge and lasting for at least three weeks, with the peak of bacterial CFU counts occurring during the first two weeks after exposure (Fig. 2A).

**Table 1:** Repartition of the animal groups (IN: Intranasal, IN+IT: Intranasal and intratracheal).

Animal ID	**Sex**	**Age at 1st exposure (months)**	**Inoculation route***exposure 1*	**Inoculation strain***exposure 1*	**Inoculation route***exposure 2*	**Inoculation strain***exposure 2*
**AA833BG**	**M**	**16**	**None**	**None**	**IN+IT**	**B1917-iGFP**
**V933BC**	**F**	**11**	**IN+IT**	**B1917-iGFP**		
**K921AD**	**M**	**11**	**IN**	**B1917-iGFP**		
**PA963A**	**F**	**13**			**None**	**None**
**PA963D**	**F**	**16**				
**K921A**	**F**	**10**	**IN**	**B1917**	**IN+IT**	**B1917**
**PA997BA**	**M**	**10**				
**V904BC**	**M**	**10**

All baboons also showed tracheal bacterial colonization, at least in the upper part of the trachea, with no major differences between groups. As previously shown for wild-type bacteria (Naninck et al., 2018; Warfel et al., 2014, 2012), animals exposed to the recombinant bacteria by the combined IN+IT route developed a typical *B. pertussis* infection, characterized by paroxysmal cough, with a peak of 20 coughs per hour during the second week of infection (Fig. 2B, blue), and leukocytosis, with a peak of 19.4 × 10^3^ cells/µL two weeks post-challenge (Fig. 2C, blue bars). Among the white blood cells (WBCs), we detected a trend towards an increase in the number of circulating lymphocytes, with a peak of 6.8 ± 2.9 × 10^3^ cells/µL (IN, B1917), 5.5 ± 1.6 × 10^3^ cells/µL (IN, B1917-iGFP), and 13.7 × 10^3^ cells/µL (IN+IT, B1917-iGFP) two weeks post-challenge (Fig. 2D). These results indicate that the pathogenicity of the modified B1917-iGFP bacteria was not affected.

On the contrary, animals challenged by the IN route only developed only mild cough, regardless of the strain used, with a maximum of 2 to 3 coughs per hour (Fig. 2B, green & gray bars). The level of circulating WBCs increased slightly, with a peak of 14.1 ± 6.6 × 10^3^ cells/µL and 9.4 ± 1.5 × 10^3^ cells/µL for baboons challenged by the IN route only with the B1917 and B1917-iGFP strains, respectively (Fig. 2C, green & gray bars). Among the WBCs, we detected a small increase in the number of circulating lymphocytes, with a peak of 6.8 ± 2.9 × 10^3^ cells/µL (IN, B1917) and 5.5 ± 1.6 × 10^3^ cells/µL (IN, B1917-iGFP) two weeks post-challenge (Fig. 2D).

We detected *B. pertussis*-specific IgG antibodies (anti-PT and anti-FHA) in the serum starting from week 2 (for anti-FHA antibodies) or week 3 (for anti-PT antibodies) post-exposure for both groups challenged with B1917-iGFP and for the group challenged with B1917, with no major differences in kinetics between the IN and IN+IT animals (Fig. 2E-F), we then considered that these animals seroconverted. Overall, these results show that B1917 and B1917-iGFP can colonize the upper respiratory tract of baboons after IN inoculation with kinetics and levels equivalent to the IN+IT inoculation route. However, we observed only mild clinical symptoms when animals were exposed by the IN route only.

### *In vivo* imaging of *B. pertussis* colonization in baboons

**Fig. 3:**
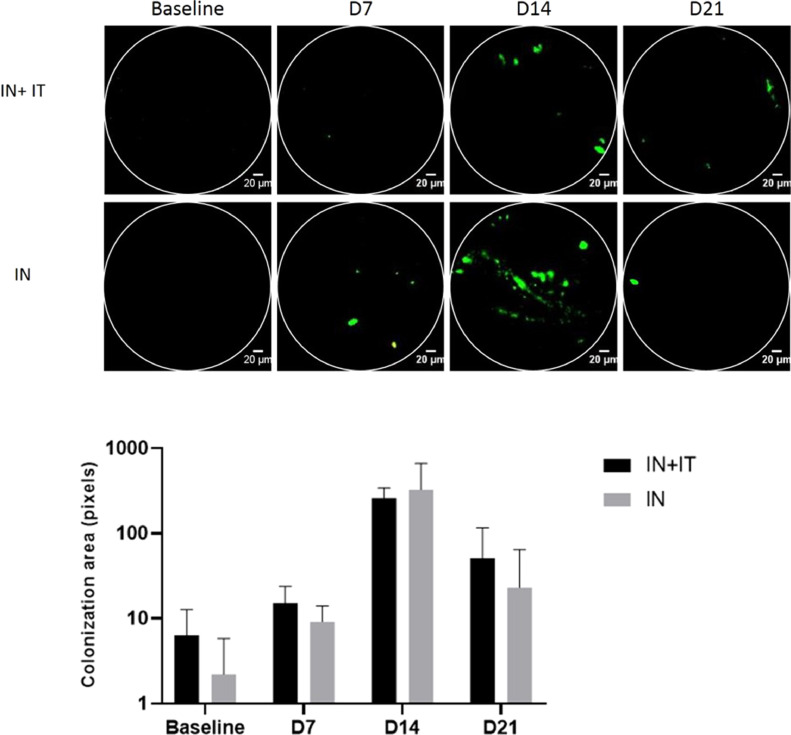
(A) *In vivo* tracheal imaging of B1917-iGFP B. pertussis (green) by pCLE imaging at baseline, and 7, 14, and 21 days post challenge. (B) Evolution of the GFP signal area in animals after intranasal (IN) or intranasal and intratracheal (IN+IT) inoculation.

We used *in vivo* bronchoscopy coupled with pCLE imaging along the trachea of naïve and *B. pertussis* B1917-iGFP-infected baboons to better characterize the features of tracheal bacterial colonization observed in animals exposed by the IN route only (Fig. 3), as previously described (Naninck et al., 2018). This *in vivo* imaging modality was reproducibly performed without affecting oximetry or the cardiac rate.

The imaging procedure allowed us to detect bacteria deeper in the trachea, which cannot be accessed using conventional swabs. We detected GFP signals, corresponding to bacterial biofilms or aggregates as already described (Naninck et al., 2018), all along the epithelia of the trachea for all infected animals starting at D7 or D14 for animals inoculated by the IN or IN+IT routes, respectively, and up to D21 post exposure (Fig. 3A)*.* There was no statistical difference (Mann Whitney t-tests) between the estimated tracheal colonization assessed by the fluorescence signal area, regardless of the inoculation route and the time post exposure (Fig. 3B).

### Infection by the in route with *B. pertussis* prevents subsequent *B. pertussis* infection

**Fig. 4:**
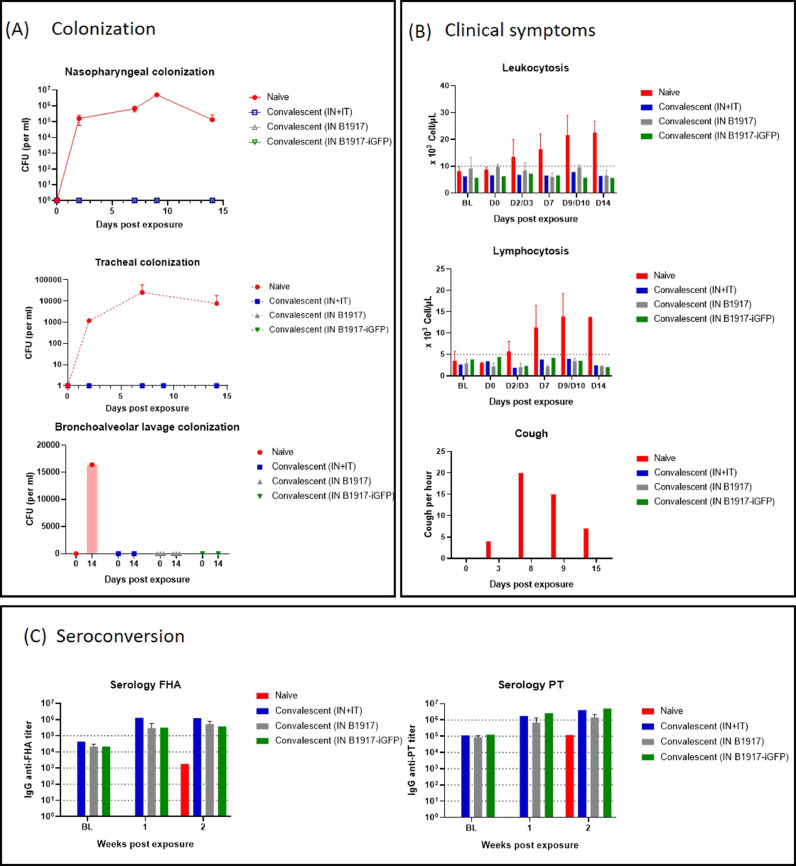
Evaluation of colonization (A) and clinical symptoms (B) after B1917 or B1917-iGFP re-infection of baboons previously exposed by the intranasal (IN) route only (gray/green bars) or the intranasal and intratracheal (IN+IT) routes (blue bars) or naïve (red bars). Nasopharyngeal, tracheal, and pulmonary B. pertussis colonization (A) was estimated by plating swabs or BAL on Bordet Gengou blood agar plates. Episodes of paroxysmal cough per hour and circulating white blood cell and lymphocyte numbers were determined over time. (C) Anti-FHA IgG and anti-PT IgG titers were determined over time.

We evaluated the effect of a first challenge on a second exposure four months after the primary infection by re-exposing convalescent baboons to the same bacterial stock of *B. pertussis* B1917-iGFP (*n* = 2) or B1917 (*n* = 3) by the IN+IT route. One naïve animal was also exposed with the same B1917-iGFP inoculum by the IN+IT route and served as a positive control (Group description in Table 1). All convalescent animals were protected from *B. pertussis* colonization of the nasopharyngeal (swabs), tracheal (swabs), and pulmonary (BAL) compartments, even baboons previously challenged by the IN route only (Fig. 4A). Only the control animal was colonized and developed classical paroxysmal cough, whereas the convalescent baboons did not cough upon the second exposure (Fig. 4B, red). Furthermore, none of the convalescent baboons showed an increase in circulating WBCs, whereas the lymphocyte numbers increased in the control animal (Fig. 4B), as expected. The second *B. pertussis* challenge was followed by similar increases (about 10-fold increase in IgG titers from the pre-reexposure levels to week 1 post re-exposure IgG titers) in serum anti-PT and anti-FHA IgG antibodies in all convalescent animals, regardless of their initial inoculation route (Fig. 4C), showing that even in the absence of detectable colonization, second exposure to *B. pertussis* boosted the antibody responses in animals primed by a first infection.

## Discussion

Recent pertussis outbreaks in several countries (“Pertussis Epidemic — California, 2014,” n.d.) have underlined the crucial need for improved prevention strategies against whooping cough. Despite high vaccination coverage, this disease still occurs in cycles every 2 to 5 years (Cherry, 1999). The pathogenesis of pertussis has been studied using a variety of animal models, including mice, rabbits, rats, guinea pigs, and newborn swine (Elahi et al., 2007, 2005; Higgs et al., 2012). However, none of these preclinical models reproduced the full spectrum of pertussis clinical symptoms, especially paroxysmal cough. The recently described baboon model of whooping cough developed by Warfel et al. [4] has the important advantage of reproducing all classical pertussis symptoms, including leukocytosis and paroxysmal cough and *B. pertussis* transmission. This model is a major breakthrough for the study of pertussis pathophysiology and provides experimental support for the hypothesis of asymptomatic carriage of *B. pertussis* in individuals vaccinated with acellular pertussis vaccines (Warfel et al., 2014). In this NHP model, animals are exposed to the pathogen by both the IN and IT routes. In humans, however, pertussis is acquired by IN exposure only and in a recently developed human challenge model of *B. pertussis* infection, solely the IN route was used to reflect natural infection (de Graaf et al., 2017). Here, we studied the effect of *B. pertussis* exposure by the IN route only in young baboons to link the NHP challenge model to the human challenge model and likely the natural infection. Juvenile baboons exposed to the pathogen by the IN route only showed colonization of the nasopharyngeal cavity and trachea, with no major differences compared to the IN+IT baboon infection model. The data were obtained by conventional swab sampling and microbiological cultures, as well as detection of GFP-expressing *B. pertussis* in the trachea by *in vivo* pCLE imaging. In contrast to the swab sampling method, this *in vivo* imaging technology also allowed us to detect the presence of bacteria in the lower respiratory tract, which may be due to progressive bacterial colonization down the airways during disease progression. This is consistent with the fact that bacteria have been found in the lungs of patients with fatal pertussis (Paddock et al., 2008). However, the presence of *B. pertussis* in the lower respiratory tract may also be due to the inoculation process, in which 1 mL of liquid bacterial suspension was introduced into the nares, as this liquid can mechanically drip down the airways. An alternative method may be to use aerosolized bacteria, as often used for mice (Misiak et al., 2017).

The inoculation of young baboons with *B. pertussis* B1917-iGFP or B1917 by the IN route only resulted in reduced clinical pertussis symptoms, with little cough and a smaller increase in leukocyte and lymphocyte counts than that observed for exposure by the IN+IT routes. This may be due to the human host-specificity of *B. pertussis* and lower virulence of this pathogen in baboons to induce pathology when exposed by the IN route only. Serological examination of the animals revealed *B. pertussis* antigen-specific antibody production starting 2 to 3 weeks following exposure by the IN route, with no major differences relative to the IN+IT inoculation model. Furthermore, re-exposure of animals by the IN+IT routes with *B. pertussis* B1917-iGFP did not lead to colonization or clinical pertussis symptoms in previously intranasally exposed baboons. These data confirm published data on the protection of animals following previous IN+IT exposure (Warfel et al., 2014). However, we additionally show that full protection can also be obtained in baboons infected by the IN route only.

Overall, our data show that exposure of young baboons to *B. pertussis* by the IN route induces specific immune responses and confers protection against subsequent *B. pertussis* exposure, at least in the short term. This inoculation model may therefore be useful for future pre-clinical evaluation of nasal candidate vaccines against pertussis.

## Author contributions

R.L.G.: Funding acquisition . C.C., R.L.G, C.L., N.R., V.C., and T.N.: Conceptualization T.N., S.L., L.C., A.HR., and M.P.: Data curation. T.N. and V.C.: Formal analysis. C.C. and R.L.G. : supervision. T.N., L.C., C.C., C.L., and R.L.G. co-wrote the manuscript. All authors reviewed and accepted the manuscript.

## Declaration of interests

Aurélie Hébert-Ribbon, Magali Pelletier and Nathalie Reveneau are Sanofi employees and may hold shares and/or stock options in the company. The other authors declare that they have no competing interests

## Declaration of Competing Interest

The authors declare no conflict of interest.

## References

[bib0001] Bart M.J., Zeddeman A., van der Heide H.G.J., Heuvelman K., van Gent M., Mooi F.R. (2014). Complete genome sequences of Bordetella pertussis isolates B1917 and B1920, representing two predominant global lineages. Genome Announc..

[bib0002] Cherry J.D. (1999). Epidemiological, clinical, and laboratory aspects of pertussis in adults. Clin. Infect. Dis. Off. Publ. Infect. Dis. Soc. Am..

[bib0003] Cole L.E., Zhang J., Pacheco K.M., Lhéritier P., Anosova N.G., Piolat J., Zheng L., Reveneau N. (2020). Immunological distinctions between acellular and whole-cell pertussis immunizations of baboons persist for at least one year after acellular vaccine boosting. Vaccines (Basel).

[bib0004] de Graaf H., Gbesemete D., Gorringe A.R., Diavatopoulos D.A., Kester K.E., Faust S.N., Read R.C. (2017). Investigating Bordetella pertussis colonisation and immunity: protocol for an inpatient controlled human infection model. BMJ Open.

[bib0005] Elahi S., Brownlie R., Korzeniowski J., Buchanan R., O'Connor B., Peppler M.S., Halperin S.A., Lee S.F., Babiuk L.A., Gerdts V. (2005). Infection of newborn piglets with Bordetella pertussis: a new model for pertussis. Infect. Immun..

[bib0006] Elahi S., Holmstrom J., Gerdts V. (2007). The benefits of using diverse animal models for studying pertussis. Trends Microbiol.

[bib0007] Hibrand-Saint Oyant L., Bourges D., Chevaleyre C., Raze D., Locht C., Salmon H. (2005). Role of Bordetella bronchiseptica adenylate cyclase in nasal colonization and in development of local and systemic immune responses in piglets. Vet. Res..

[bib0008] Higgs R., Higgins S.C., Ross P.J., Mills K.H.G. (2012). Immunity to the respiratory pathogen Bordetella pertussis. Mucosal. Immunol..

[bib0009] Inatsuka C.S., Xu Q., Vujkovic-Cvijin I., Wong S., Stibitz S., Miller J.F., Cotter P.A. (2010). Pertactin is required for Bordetella species to resist neutrophil-mediated clearance. Infect. Immun..

[bib0010] Jahnmatz M., Richert L., Al-Tawil N., Storsaeter J., Colin C., Bauduin C., Thalen M., Solovay K., Rubin K., Mielcarek N., Thorstensson R., Locht C., BPZE1 study team (2020). Safety and immunogenicity of the live attenuated intranasal pertussis vaccine BPZE1: a phase 1b, double-blind, randomised, placebo-controlled dose-escalation study. Lancet Infect. Dis..

[bib0011] Locht C. (2017). Will we have new pertussis vaccines?. Vaccine.

[bib0012] Locht C. (2016). Live pertussis vaccines: will they protect against carriage and spread of pertussis?. Clin. Microbiol. Infect..

[bib0013] Locht C., Geoffroy M.C., Renauld G. (1992). Common accessory genes for the Bordetella pertussis filamentous hemagglutinin and fimbriae share sequence similarities with the papC and papD gene families. EMBO J.

[bib0014] Menozzi F.D., Mutombo R., Renauld G., Gantiez C., Hannah J.H., Leininger E., Brennan M.J., Locht C. (1994). Heparin-inhibitable lectin activity of the filamentous hemagglutinin adhesin of Bordetella pertussis. Infect. Immun..

[bib0015] Merkel T.J., Halperin S.A. (2014). Nonhuman primate and human challenge models of pertussis. J. Infect. Dis..

[bib0016] Misiak A., Wilk M.M., Raverdeau M., Mills K.H.G. (2017). IL-17-producing innate and pathogen-specific tissue resident memory γδ T cells expand in the lungs of Bordetella pertussis-infected mice. J. Immunol. Baltim. Md.

[bib0017] Naninck T., Coutte L., Mayet C., Contreras V., Locht C., Le Grand R., Chapon C. (2018). *In vivo* imaging of bacterial colonization of the lower respiratory tract in a baboon model of Bordetella pertussis infection and transmission. Sci. Rep..

[bib0018] Paddock C.D., Sanden G.N., Cherry J.D., Gal A.A., Langston C., Tatti K.M., Wu K.-.H., Goldsmith C.S., Greer P.W., Montague J.L., Eliason M.T., Holman R.C., Guarner J., Shieh W.-.J., Zaki S.R. (2008). Pathology and pathogenesis of fatal Bordetella pertussis infection in infants. Clin. Infect. Dis..

[bib0019] Pertussis Epidemic — California, 2014 [WWW Document], n.d. URL https://www-cdc-gov.gate2.inist.fr/mmwr/preview/mmwrhtml/mm6348a2.htm (accessed 9.6.17).

[bib0020] Thiberville L., Moreno-Swirc S., Vercauteren T., Peltier E., Cavé C., Bourg Heckly G. (2007). In vivo imaging of the bronchial wall microstructure using fibered confocal fluorescence microscopy. Am. J. Respir. Crit. Care Med..

[bib0021] Warfel J.M., Beren J., Kelly V.K., Lee G., Merkel T.J. (2012). Nonhuman primate model of pertussis. Infect. Immun..

[bib0022] Warfel J.M., Zimmerman L.I., Merkel T.J. (2014). Acellular pertussis vaccines protect against disease but fail to prevent infection and transmission in a nonhuman primate model. Proc. Natl. Acad. Sci. U. S. A..

[bib0023] WHO | World Health Organization [WWW Document], n.d. . WHO. URL http://www.who.int/immunization/monitoring_surveillance/en/ (accessed 9.29. 2020).

[bib0024] Yeung K.H.T., Duclos P., Nelson E.A.S., Hutubessy R.C.W. (2017). An update of the global burden of pertussis in children younger than 5 years: a modelling study. Lancet Infect. Dis..

